# Circulating Extracellular Vesicles in Cardiovascular Disease

**DOI:** 10.3390/ijms26146817

**Published:** 2025-07-16

**Authors:** Ilenia Pia Cappucci, Elena Tremoli, Barbara Zavan, Letizia Ferroni

**Affiliations:** 1 Maria Cecilia Hospital, GVM Care and Research, 48033 Cotignola, Italy; ilenia.cappucci@unife.it (I.P.C.); tremoli@gvmnet.it (E.T.); 2 Translational Medicine Department, University of Ferrara, 44121 Ferrara, Italy

**Keywords:** extracellular vesicles, exosomes, microparticles, miRNA, atherosclerosis, myocardial infarction, heart failure, hypertension, biomarkers, cardiovascular disease

## Abstract

Despite notable advancements in clinical care, cardiovascular disease (CVD) remains a leading global cause of mortality. Encompassing a wide range of heart and blood vessel disorders, CVD requires targeted prevention and treatment strategies to mitigate its public health impact. In recent years, extracellular vesicles (EVs) have emerged as crucial mediators of intercellular communication, influencing key processes such as vascular remodeling, inflammation, and immune responses in CVDs. EVs, including exosomes and microvesicles, carry bioactive molecules such as miRNAs, proteins, and lipids that contribute to disease progression. They are released by various cell types, including platelets, erythrocytes, leukocytes, endothelial cells, and cardiomyocytes, each playing distinct roles in cardiovascular homeostasis and pathology. Given their presence in circulating blood and other body fluids, EVs are increasingly recognized as promising non-invasive biomarkers for CVD diagnosis and prognosis. Furthermore, EV-based therapeutic strategies, including engineered EVs for targeted drug delivery, are being explored for treating atherosclerosis, myocardial infarction, heart failure, and hypertension. However, challenges remain regarding the standardization of EV isolation and characterization techniques, which are critical for their clinical implementation. This review highlights the diverse roles of EVs in CVD pathophysiology, their potential as diagnostic and prognostic biomarkers, and emerging therapeutic applications, clearing the way for their integration into cardiovascular precision medicine.

## 1. Introduction

Cardiovascular diseases (CVDs) encompass a range of conditions affecting the heart and blood vessels, including coronary heart failure, peripheral artery disease, and vascular disease, among others [1,2,3]. In the early twenty-first century, CVDs stood as the leading cause of premature mortality and morbidity worldwide [4].

The escalating focus on CVDs as a global epidemic has driven increased public awareness and initiatives to enhance overall health. The American Heart Association promotes seven ideal health metrics, encompassing both health behaviors (such as non-smoking, physical activity, maintaining a healthy body mass index (BMI), and adhering to a nutritious diet) and health factors (including blood sugar, blood pressure, and total cholesterol [5]). Despite advancements in cardiovascular treatments, challenges such as unreliable outcomes and increased treatment costs persist for chronic CVD cases [5]. Unfortunately, therapeutic interventions for CVDs typically begin after the onset of clinical symptoms, aimed at symptom elimination. Consequently, the implementation of early pathogenetic therapy for CVD remains a critical task in modern research and healthcare [6].

Currently, in clinical practice, there are various circulating markers to diagnose the onset and prognosis of CVDs currently used in clinical practice (Table 1).

Among all, the most investigated are cardiac troponin I and T (cTn-I and cTn-T) and cardiac natriuretic peptides (CNPs). cTn-I and cTn-T are isoforms exclusive to cardiac myocytes, and their measurement is highly sensitive and specific for detecting cardiac injury [14,15]. Prolonged ischemia of the myocardium, often induced by myocardial infarction (MI), is a prevalent cause of necrosis in heart muscle cells; proteins such as cardiac troponin from deceased cardiomyocytes migrate into the bloodstream, where they become detectable through various immunochemical methods [16]. The determination of cardiac troponin concentrations in serum is valuable for identifying cardiomyocyte damage associated with conditions such as pulmonary embolism, inflammatory heart diseases (endocarditis, myocarditis, pericarditis), sepsis, and chronic renal failure [17,18]. Given that elevated cardiac troponin levels correlate with the extent of myocardial damage in these conditions and hold prognostic significance, incorporating this additional diagnostic method can enhance existing algorithms for managing patients with pulmonary embolism, inflammatory heart diseases, sepsis, and chronic renal failure [17].

Atrial natriuretic peptides (ANPs) and brain natriuretic peptides (BNPs), also known as B-type peptides, stand out as the most pertinent natriuretic peptide (NP) biomarkers for both the diagnosis and prognosis of heart failure (HF), as well as the assessment of underlying CVD often linked to heightened ventricular stress and congestive disorders [19]. CNPs undergo a synthesis process as precursor proteins. They are then subjected to intracellular modification to form prohormones (pro-ANP and pro-BNP) and are subsequently cleaved into their active forms. Under normal physiological conditions, pro-ANP is primarily expressed by atrial tissue. The secretion of ANP occurs in response to the stretching of the atrial walls and serves two primary purposes: vasodilation and increasing the renal excretion of sodium and water [19]. However, providing the serum amount of ANP is problematic because of its short half-life (2–5 min) due to its quick cleavage by neprilysin [20]. Instead, circulating BNP and N-terminal pro-B-type natriuretic peptide (NT-proBNP) levels are typically low under normal conditions but have a significant increase in HF as part of a compensatory mechanism to restore normal hemodynamics. BNPs facilitate arterial vasodilation, diuresis, and natriuresis. Moreover, they exert anti-hypertrophic and anti-fibrotic effects, while counteracting the activation of the renin–angiotensin–aldosterone system (RAAS), sympathetic nervous system (SNS), and the endothelin systems [21].

Despite the significant impact of clinical diagnostic markers such as cardiac troponins and cardiac natriuretic peptides on the diagnosis and management of CVDs, their substantial limitations persist. Indeed, the remarkable sensitivity of cTn-I and cTn-T assays is offset by their restricted diagnostic specificity. Moreover, factors like age and non-CVD-related complications such as renal dysfunction can confound the plasma concentration of B-type natriuretic peptides. Given these limitations, additional diagnostic modalities are often required to provide a more comprehensive assessment of CVDs. For example, one of the most prevalent and life-threatening CVDs is atherosclerosis, which is primarily assessed through imaging techniques such as carotid intima–media thickness measurement via ultrasound, coronary angiography, computed tomography angiography, and magnetic resonance angiography. These methods help evaluate vascular structure and plaque burden, but they are often invasive, costly, or not readily accessible [22].

Similarly, hypertension—a major risk factor for atherosclerosis and other cardiovascular conditions—is diagnosed through repeated blood pressure measurements using sphygmomanometry or ambulatory blood pressure monitoring to confirm persistently elevated values. While effective, these approaches rely on indirect indicators and may not capture early disease progression.

The absence of highly specific biomarkers limits early detection and risk stratification, making it challenging to intervene before significant disease progression occurs. This highlights the urgent need for novel molecular targets that could refine non-invasive diagnostic approaches. Advancing biomarker research could bridge existing gaps, improve diagnostic accuracy, and enable earlier risk assessment, ultimately leading to better patient outcomes in CVDs [23].

In the realm of cell biology, extracellular vesicles (EVs) have emerged as a significant innovation in recent decades, playing a crucial role in enhancing intercellular communication [24]. Actively released from cells, EVs serve as carriers for bioactive cargo, including enzymes, cytokines, and miRNAs. These molecular components are delivered to target cells via paracrine signaling or circulation [25,26]. This review focuses on the complex relationship between EVs and CVDs, highlighting their potential as biomarkers for diagnosis and as strategic therapeutic agents.

## 2. Extracellular Vesicles

The International Society for Extracellular Vesicles defines EVs as lipid bilayer membrane vesicles with a diameter of less than 1 micrometer released by various cells and incapable of replicating them on their own [27]. Produced by nearly all cell types in the human body, EVs are ubiquitous in various body fluids, including blood, urine, seminal fluid, breast milk, and saliva [23]. The EV population includes vesicles of variable size and can be classified based into small EVs (<200 nm in diameter) and large EVs (>200 nm in diameter). Based on their biogenesis, EVs can be namely exosomes, microvesicles, and apoptotic bodies [27].

**Figure 1:**
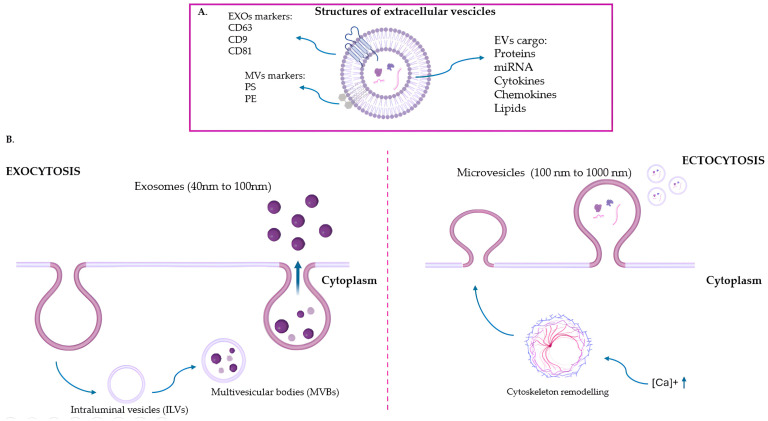
Extracellular vesicles (EVs). (**A**) EVs serve as carriers for bioactive cargo, including enzymes, cytokines, chemokines, miRNAs, and lipids. On the surface, exosomes (EXOs) express the specific tetraspanins CD63, CD9, and CD81, while microvesicles (MVs) present phosphatidylserine (PS) and phosphatidylethanolamine (PE). (**B**) EXOs are generated by exocytosis: the Endosomal Sorting Complexes Required for Transport (ESCRT), along with auxiliary proteins (Alix, VPS4, VTA-1), recognize ubiquitination-modified proteins and screen specific molecules into exosomal precursors enabling the formation of intraluminal vesicles (ILVs). Then, ESCRT-III complex is required for the formation of spirals, which cause inward budding and vesicle fission to produce multivesicular bodies (MVBs). When MVBs fuse with the plasma membrane, exosomes are discharged into the extracellular environment. In contrast, MVs are formed by ectocytosis, i.e., the outward budding of the plasma membrane. This process is facilitated by a calcium-dependent mechanism that induces cytoskeletal alterations on the plasma membrane. External signals cause an increase in intracellular calcium, which disrupts asymmetry in the double phospholipid layer by influencing the activity of various enzymes such as flippases (inward-directed pumps), floppases (outward-directed pumps), and scramblases (enzymes that promote unspecific bidirectional redistribution across the bilayer). Calcium ions also play a role in the activation of enzymes such as gelsolin and calpain, which disassemble the actin cytoskeleton. This alteration in the actin cytoskeleton influences the curvature and protrusion of the plasma membrane, facilitating the detachment of microvesicles from the membrane.

Exosomes, which range from 40 to 100 nanometers, have high levels of surface markers like CD63, CD9, and CD81 and contain proteins that help them biosynthesize, such as Alix, TSG101, and FLOT-1 [26,27] (Figure 1). Exosomes are intraluminal vesicles (ILVs) that originate from the inward budding of the endosomal membrane during multivesicular body (MVB) maturation. Briefly, the Endosomal Sorting Complexes Required for Transport (ESCRT), along with auxiliary proteins (Alix, VPS4, VTA-1), recognize ubiquitination-modified proteins and screen specific molecules into exosomal precursors. As a result, they enable the formation of ILVs and their fission to produce MVBs [28]. When MVBs fuse with the plasma membrane, vesicles are discharged into the extracellular environment; hence, they are called exosomes. The specific molecular mechanism for MVB fusion with the plasma membrane is not well understood; however, soluble N-ethylmaleimide-sensitive fusion attachment protein receptor (SNARE), tethering factors, RAB proteins, and RAB GTPases are implicated in membrane fusion [29].

Microvesicles, also known as microparticles or ectosomes, range in size from 100 nm to 1000 nm (Figure 1). They are formed through the outward budding of the plasma membrane through a calcium-dependent mechanism that induces actin cytoskeleton alteration, which generates curvature and protrusion of the plasma membrane [30]. The content of microvesicles reflects the features and activation status of the parent cell [31]. Identified by expression of phosphatidylserine (PS) and phosphatidylethanolamine (PE) on their surfaces, microvesicles can be indicative of release from apoptotic or activated cells. The selective binding of annexin to PS can be utilized to detect the exposed PS on the surface of apoptotic cells and the subclass of PS-positive microvesicles. Indeed, not all microvesicles are positive. Some microvesicles also exhibit positivity for markers such as CD40 ligand and Annexin A1 [28,31]. The presence of these markers adds to the heterogeneity of microvesicles populations, reflecting diverse cellular origins and functions. However, further research is needed to gain a better understanding of the different markers in microvesicles, as this knowledge is crucial to elucidate their role in cellular communication, disease mechanisms, and potential therapeutic applications.

Apoptotic bodies are formed from the plasma membrane as blebs when cells die [32]. Apoptotic membrane bleb production is a late step of programmed cell death regulated by caspase-mediated cleavage and subsequent activation of Rho-associated protein kinases. They include similar intracellular fragments and cellular organelles to microvesicles, such as histones, DNA fragments, degraded proteins, nuclear fractions, coding RNAs, noncoding RNAs, and DNAs, but are typically 50 to 5000 nm in diameter [32].

## 3. Extracellular Vesicles in Bloodstream

Bloodstream EVs can originate from almost any cell or tissue in the human body. Erythrocytes, platelets, leukocytes, vascular cells, and heart cells are the primary cell types capable of releasing EVs [31,33]. Table 2 summarizes some of the reported markers used to distinguish the origin of bloodstream EVs.

For the purpose of clarity, we have used the term EVs in this review to encompass both exosomes and microvesicles. This decision was made because many studies do not consistently distinguish between these subtypes and the distinction can be difficult due to overlapping characteristics such as size, biogenesis, and function. By using EVs as an overarching term, we aim to capture the broader spectrum of vesicles reported in the literature, regardless of the specific classification used in individual studies.

Platelet-derived EVs are notably abundant in plasma due to the high numbers of platelets in the bloodstream [42]. These EVs are characterized by markers specific to platelets, such as CD41a, CD61, and GPIb [40].

As described by Terrisse et al. [43], platelet-derived EVs exhibit procoagulant activity. Indeed, they adhere to endothelial cells via lactadherin, PS, and the αVβ3 integrin. This interaction stimulates the production of reactive oxygen species (ROS) in endothelial cells, leading to increased expression of von Willebrand factor (VWF) on the endothelial surface. The elevated VWF facilitates the binding of circulating platelets via GPIb and P-selectin, promoting platelet adhesion and clot formation [43]. Additionally, platelet-derived EVs exert an anti-inflammatory influence by downregulating macrophage activation and consequently reducing the release of cytokines such as TNF-α and IL-10 [44]. Moreover, platelet-derived EVs influence the differentiation of monocytes into immature dendritic cells (iDCs) and their subsequent maturation into fully functional dendritic cells (DCs). This interference disrupts the normal progression of immune responses by reducing the expression of key surface markers like HLA molecules and the costimulatory molecule CD80 on iDCs [44]. The platelet-derived EVs are also recognized for their significant angiogenic potential, which is largely attributed to their protein cargo [45]. These vesicles carry crucial growth factors, such as platelet-derived growth factor (PDGF), fibroblast growth factor 2 (FGF2), and vascular endothelial growth factor (VEGF), as well as other potential lipid growth factors. This rich composition enables platelet-derived EVs to promote cell mobilization and migration, thereby facilitating angiogenesis and contributing to tissue repair and regeneration. These properties make platelet-derived EVs key players in processes like wound healing and vascular remodeling [45].

Erythrocytes generate EVs that play a crucial role in expelling harmful substances and facilitating the premature elimination of themselves from the bloodstream [36]. These vesicles are characterized by low levels of glyceraldehyde-3-phosphate dehydrogenase (GAPDH), a protein known for its high affinity for the cytoplasmic domain of band 3. This downregulation promotes the aggregation of band 3 and contributes to the formation of senescent cell-specific antigens. Erythrocyte-derived EVs are rich in various proteins, including ALIX, TSG101, and CD63, alongside erythrocyte-specific proteins such as actin, hemoglobin A, CD55, CD59, and CD235a, reflecting their cellular origin [36]. Gao et al. [46] explored the activity of erythrocyte-derived EVs, showing that these EVs could moderately increase the mRNA and protein levels of pro-inflammatory cytokines, such as TNF-α, IL-6, and IL-1β, in macrophages [46]. Functional studies revealed that the pro-inflammatory effects of erythrocyte-derived EVs were mediated through the TLR4-MyD88-NF-κB and MAPK signaling pathways [46]. Moreover, erythrocyte-derived EVs promote the polarization of macrophages towards a pro-inflammatory state, as evidenced by the upregulation of pro-inflammatory markers like inducible nitric oxide synthase (iNOS), TNF-α, and IL-6, as well as enhanced phagocytic activity [46]. However, exact components within the EVs responsible for macrophage polarization remain unidentified [46].

EVs derived from immune cells are crucial mediators of intercellular communication, regulating specific pathways in both adaptive and innate immune responses [37]. Leukocyte-derived EVs express adhesion molecules such as PSGL-1, CD11b, ICAM1, and IL-1. In addition to IL-1, these EVs transport active caspase 1, an enzyme connected to the inflammasome machinery responsible for cleaving pro-IL-1 and pro-IL-18 into their bioactive secretory forms. Functioning as paracrine messengers, EVs exert influence on the innate immune system in the context of infections, sepsis, and chronic inflammatory disorders like rheumatoid arthritis, atherosclerosis, and type 2 diabetes [37]. Notably, EVs from distinct leukocyte subpopulations exhibit variations in the composition of plasma membrane and cytosolic proteins. For instance, monocyte-derived EVs express CD11b, CD14, CD64, and CD142, while lymphocyte-derived EVs carry CD3 and CD45. Neutrophil-derived EVs, on the other hand, contain CD35, CD66b, and myeloperoxidase (MPO) [37,39].

Vascular endothelial cells, which are highly prevalent in well-perfused tissues, serve as frontline responders to hypoxia, playing a pivotal role in paracrine signaling also through the release of EVs [47]. The inclusion of antioxidants, heat-shock proteins, unfolded protein response intermediates, and endoplasmic reticulum-resident chaperones suggests their involvement in preserving cellular health. Notably, these EVs aid in angiogenesis and cell migration by delivering miR-214, which inhibits the ATM pathway, a crucial regulator of DNA damage response and cell cycle progression. By suppressing this pathway, miR-214 helps prevent cellular senescence and promotes blood vessel formation [48]. Additionally, endothelial-derived EVs contain important proteins such as glycolytic enzymes, AMPK signaling intermediates, and ribosomal proteins like Rps3a, which are involved in regulating apoptosis. These proteins boost glycolytic activity and ATP production, essential for maintaining energy balance and supporting cardiac function during and after ischemic events [47]. Moreover, endothelial cell vesicles sustain a chronic state of vascular activation, facilitating the expression of adhesion molecules such as ICAM-1 and VCAM-1 and releasing pro-inflammatory cytokines. This leads to endothelial dysfunction, microvascular damage, and a pro-thrombotic environment that intensifies arterial and venous thrombotic events [49].

Finally, cardiomyocyte-derived EVs expose caveolin-3 and flotillin-1 on their surface and transport sarcomeric and mitochondrial proteins, including tropomyosin, myosin, cardiac-type myosin-binding protein C (MYBPC3), and valosin-containing protein (VCP) [34]. Notably, heat-shock proteins such as Hsp20, Hsp60, and Hsp70 are abundant in these EVs, playing crucial roles in promoting cardiomyocyte growth and survival [50]. For instance, Hsp20 has angiogenic activity, inducing the expression of vascular endothelial growth factor (VEGF) receptor-2 (VEGFR2) in endothelial cells that stimulate cardiac angiogenesis in response to hypoxia [51]. Instead, Tian et al. [52] found Hsp60 abnormally translocated to cardiomyocyte plasma membranes and the extracellular space after MI via EVs, potentially activating TLR2 and TLR4, contributing to myocardial inflammation [52]. Finally, Hsp70 activates monocytes, leading to their adhesion to endothelial cells, a process that may contribute to the development of atherosclerosis. Hsp70 could act as a pro-inflammatory cytokine, potentially exacerbating vascular disease [53]. Additionally, these EVs transport inflammatory factors such as IL-6 and TNF-α, contributing to cardiac remodeling. Within cardiac cell-derived EVs, biomolecules like GLUT4, GLUT1, and lactate dehydrogenase are present [54]. When these vesicles are taken up by endothelial cells, they enhance glucose uptake and pyruvate production in these cells, potentially providing nutritional support to the cardiomyocytes [54].

## 4. EVs as Possible Novel Biomarkers for CVDs

**Figure 2:**
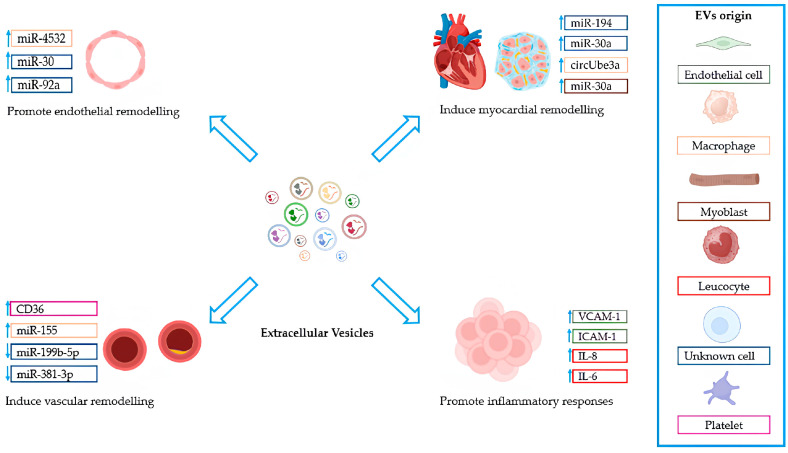
The multifaceted roles of EVs in cardiovascular remodeling and inflammation. EVs have demonstrated the ability to transport biological content to target endothelial cells, smooth vascular muscle cells, and atherosclerotic plaques, thereby influencing vascular function and the progression of CVDs. The panel on the right lists the main cellular sources of EVs involved in these processes.

Typically, an ideal biomarker should (i) be accessible through non-invasive methods; (ii) demonstrate high sensitivity and specificity for the disease; (iii) be detectable in the early stages; (iv) show sensitivity to changes in the disease; (v) possess stability, indicated by a long half-life within the sample; and (vi) be accurately and rapidly detectable [55]. EVs have demonstrated the ability to transport biological content to target endothelial cells, vascular smooth muscle cells, and atherosclerotic plaques, thereby influencing vascular function and the progression of CVDs (Figure 2 and Table 3).

Consequently, the association between circulating EV levels and major adverse cardiac events has been established [55]. The non-invasive nature of collecting circulating EVs allows for the acquisition of biological data at any time, offering insights into vascular or myocardial homeostasis and specific disease states for epidemiological studies. Moreover, the bioavailability of EVs in various body fluids facilitates longitudinal investigations, eliminating the need for invasive coronary diagnostics or radiographic tests [65]. Despite the promising potential of using EV counts from specific cell types as biomarkers, a significant challenge lies in the absence of standardized techniques [65].

### 4.1. EVs and Atherosclerosis

Atherosclerosis (AS) is a chronic inflammatory disease characterized by the accumulation of lipids, fibrous debris, and calcium within the inner layers of medium- and large-sized arteries. This pathological process leads to the formation of plaques that stiffen and narrow arterial walls, restricting blood flow and significantly increasing the risk of cardiovascular complications [66]. Atherosclerosis starts with LDL infiltration and oxidation in the arterial wall, forming oxidized LDL (Ox-LDL). This triggers immune responses, recruiting monocytes that differentiate into macrophages and form foam cells. Scavenger receptors (CD36, SR-A) enhance inflammation and monocyte recruitment. Oxidative stress damages the endothelium, upregulating adhesion molecules (ICAM-1, VCAM-1) and promoting leukocyte adhesion and plaque formation [67,68]. These inflammatory pathways contribute to the formation of fatty streaks, which later transform into fibrous plaques with a necrotic lipid-rich core [69]. With the progression of the AS, fibrous plaques may destabilize and rupture, leading to thrombosis. This process can result in severe clinical events such as myocardial infarction [70].

Emerging evidence underscores the complex role of EVs in modulating inflammatory processes central to AS. Notably, platelet-derived EVs have been found to exhibit high levels of ubiquitination, a modification that accelerates the degradation of CD36 [71]. This receptor plays a pivotal role in platelet adhesion to collagen in blood vessels, a critical step in thrombus formation and hemostasis. The loss of CD36 function not only affects platelet aggregation but also inhibits lipid and particle accumulation in macrophages. Since CD36 is responsible for the uptake of Ox-LDL by macrophages, its suppression reduces foam cell formation, thereby influencing plaque development [71].

Endothelial cell-derived EVs also contribute significantly to AS progression by modulating immune cell recruitment and vascular inflammation. Studies have shown that EVs isolated from the endothelial cells of AS patients exhibit markedly increased expression of ICAM-1, a key adhesion molecule involved in the recruitment of leukocytes to sites of vascular injury and inflammation. This recruitment is crucial for the initiation and advancement of coronary artery disease [58]. Additionally, endothelial-derived EVs carry a range of pro-inflammatory mediators, including chemotactic cytokines such as IL-6, IL-8, CXCL-10, and monocyte chemoattractant protein-1 (CCL-2) [57]. The presence of these cytokines within EVs contributes to the early stages of AS by promoting endothelial dysfunction, enhancing monocyte/macrophage adhesion to the endothelium, and facilitating their migration into the intima [57]. Once macrophages internalize Ox-LDL, they differentiate into foam cells. This transformation is further exacerbated by macrophage-derived EVs, which alter miRNA cargo [68].

Research by He et al. [59] demonstrated that Ox-LDL exposure leads to increased levels of miR-155 in EVs, which are subsequently transferred to monocytes. This transfer promotes a phenotypic shift from anti-inflammatory M2 macrophages to pro-inflammatory M1 macrophages, intensifying inflammatory responses and worsening lesion formation [59]. Furthermore, Ox-LDL induces macrophages to differentiate into foam cells by upregulating miR-4532 expression [56]. This particular miRNA targets and inhibits the transcription factor Sp1, a regulator of cell differentiation, proliferation, and apoptosis. The suppression of Sp1 subsequently activates the NF-κB-P65 signaling pathway, a major regulator of inflammatory responses. The activation of this pathway enhances the expression of adhesion molecules such as ICAM-1 and VCAM-1 in endothelial cells, further contributing to endothelial dysfunction [56].

Building on the understanding of miRNA involvement in AS, studies by Blaser et al. [72] have shown that miR-199b-5p and miR-381-3p levels decreased in plaque-derived EVs compared to normal controls. The reduction in miR-199b-5p is associated with the production of reactive oxygen species, mitochondrial fission, and endothelial apoptosis. In contrast, miR-381-3p plays a protective role by mitigating endothelial inflammatory damage caused by Ox-LDL while also suppressing oxidative stress and hyperglycemia-induced smooth muscle cell proliferation. The imbalance caused by the downregulation of these miRNAs contributes to endothelial dysfunction and increased oxidative stress, both of which accelerate the progression of atherosclerotic lesions and promote plaque instability, ultimately increasing the risk of cardiovascular events [72].

In addition, EVs from patients with coronary AS contain elevated levels of miR-30 and miR-92a, which target the ATP-binding cassette transporter A1 (ABCA1) [60]. These proteins are responsible for the transfer of phospholipids and cholesterol to high-density lipoprotein cholesterol (HDL-C), a process crucial for cholesterol homeostasis. In addition to its role in lipid metabolism, ABCA1 regulates key vascular functions, including endothelial homeostasis, blood pressure control, and platelet aggregation. The dysregulation of ABCA1 by miR-30 and miR-92a within EVs exacerbates vascular inflammation, disrupts lipid transport, and enhances platelet synthesis and aggregation, further fueling the progression of AS [60].

With the advancement of AS, plaques become vulnerable to rupture, a process exacerbated by the secretion of matrix metalloproteinases (MMPs) from foam cells and EVs. The degradation of extracellular matrix components exposes the necrotic lipid core, triggering platelet aggregation and thrombus formation. Platelet-derived EVs, enriched in tissue factor and P-selectin, enhance coagulation and promote the formation of occlusive thrombi, leading to severe cardiovascular events [73].

### 4.2. EVs and Myocardial Infarction

MI is caused by the rupture of unstable atherosclerotic plaques, leading to coronary thrombosis and subsequent myocardial ischemia/reperfusion injury. Despite therapeutic advances, acute MI remains the leading cause of cardiovascular morbidity and mortality worldwide [74]. It originates from AS, where fatty plaques accumulate in coronary arteries, narrowing them and limiting blood flow. Plaque rupture exposes its contents, triggering thrombus formation that can partially or completely occlude the artery, cutting off oxygen supply to the myocardium. Oxygen deprivation impairs ATP production, leading to cell injury and, if prolonged, myocardial necrosis. This cascade underlies the clinical manifestations and complications of MI [75].

Circulating EVs influences the inflammatory response after MI by interacting with the inflammatory marker C-reactive protein (CRP) [76]. These EVs have been shown to bound and induce conversion of pentameric CRP (pCRP) to the pro-inflammatory monomeric form (mCRP), which then binds to endothelial cells and enhances the inflammatory response after MI [76]. In addition, a study by van der Zee et al. [77] showed that EVs bound with CRP and IgG are associated with the activation of complement components such as C1q, C3, and C4. This suggests that in acute MI, complement activation is particularly driven by this EV-bound protein. The complement cascade is further amplified through the formation of the membrane attack complex (MAC), which exacerbates endothelial damage and increases vascular permeability, worsening myocardial injury [77]. Beyond inflammation, EVs also influence myocardial fibrosis and remodeling through the transfer of noncoding RNAs to cardiac fibroblasts [78]. Macrophage-derived EVs play a particularly significant role in this process. For example, EVs carrying circular RNA circUbe3a from M2 macrophages exacerbate myocardial fibrosis by modulating cardiac fibroblast proliferation, migration, and phenotype through the miR-138–5p/RhoC axis [78]. Conversely, activated M1 macrophages transfer miR-155-loaded EVs to cardiac fibroblasts, suppressing fibroblast proliferation by downregulating the Son of Sevenless gene (Sos1) and promoting inflammation through decreased expression of the anti-inflammatory gene Suppressor of Cytokine Signaling 1 (Socs1) [78,79]. The deleterious effects of miR-155 extend to endothelial function as well, as shown by Liu et al. [80] who demonstrated that miR-155 impairs angiogenesis by targeting the RAC1-PAK1/2 and Sirt1/AMPK-eNOS pathways, both of which are essential for vascular repair following MI [80].

Autophagy, a critical cellular process for maintaining cardiomyocyte survival under stress, is also regulated by EVs in the context of MI. The dynamic regulation of autophagy is particularly important in cardiomyocytes experiencing hypoxia. Indeed, recent research by Yang et al. [62] has highlighted the dynamic regulation of autophagy in cardiomyocytes under hypoxia, identifying miR-30a as a key regulator. Their study shows that autophagy in cardiomyocytes peaked at 2 h of hypoxia and then decreased, with miR-30a playing a key role in this process. MiR-30a directly targets Beclin-1 and ATG5, essential components of autophagy machinery, thereby inhibiting autophagy [62]. These findings suggest that EV-mediated miR-30a transfer may contribute to impaired cardiomyocyte survival and increased susceptibility to MI-induced damage.

Additionally, EVs serve as carriers of key proteins implicated in MI pathophysiology. A study by de Hoog et al. [64] identified three overexpressed proteins in serum-derived EVs from MI patients: polymeric immunoglobulin receptor (pIgR), cystatin C, and complement factor C5a. pIgR, a protein primarily involved in mucosal immunity, facilitates IgA transport and indirectly enhances immune function. Cystatin C, a cysteine proteinase inhibitor widely recognized as a marker of kidney function, has also been linked to CVD independently of renal impairment, though its specific diagnostic value for acute myocardial ischemia remains unclear. Finally, complement factor C5a, a potent anaphylatoxin, plays a critical role in inflammation. It triggers inflammatory responses by recruiting neutrophils and macrophages to sites of injury or infection. C5a is particularly important in the early stages of inflammation following MI. Finally, complement factor C5a, a potent anaphylatoxin, plays a crucial role in post-MI inflammation by recruiting neutrophils and macrophages to the site of injury. C5a contributes to both tissue repair and reperfusion injury, and elevated plasma levels of C5a have been associated with an increased risk of future cardiovascular events in patients with advanced AS, highlighting its prognostic potential [64]. At the molecular level, the interaction between EVs and myocardial cells is largely mediated by surface receptors and intracellular signaling pathways. For instance, integrin-mediated EV uptake by endothelial cells triggers the NF-κB signaling pathway, leading to the upregulation of pro-inflammatory cytokines such as TNF-α and IL-6 [81]. Moreover, EV-mediated delivery of heat-shock proteins (HSP70 and HSP90) has been shown to activate Toll-like receptors (TLRs), particularly TLR4, further amplifying the inflammatory response and myocardial damage [81].

### 4.3. EVs and Heart Failure

HF is a condition in which the heart cannot pump blood effectively, resulting in insufficient oxygen and nutrient delivery to organs and tissues [82]. Loss of myocytes and increased mechanical strain lead the remaining myocytes to undergo hypertrophy. This process, along with direct injury and neurohormonal activation, contributes to fibrosis and progressive dilation of the left ventricle, altering its shape from an elliptical to a more spherical form [83]. This remodeling raises myocardial oxygen demand and reduces contractile efficiency. As dilation progresses, impaired pumping leads to increased preload and afterload, worsening cardiac output and causing symptoms like weakness, dyspnea, and fluid retention. Additionally, altered ventricular geometry and functional mitral regurgitation further compromise cardiac function, perpetuating a vicious cycle of heart failure progression [84].

Mitochondrial dysfunction plays a central role in HF progression, as impaired ATP production and excessive ROS generation lead to cellular damage, apoptosis, and further weakening of myocardial function. Chronic neurohormonal activation, particularly of the RAAS, exacerbates fibrosis and hypertrophy. Additionally, cytoskeletal remodeling and extracellular matrix alterations, driven by elevated MMPs and integrin dysfunction, compromise myocardial integrity, worsening HF progression and limiting cardiac recovery [61]. Different EV subpopulations, particularly miRNA-enriched EVs, contribute significantly to this dysregulation. A study by Matsumoto et al. [61] demonstrated that miR-192, miR-194, and miR-34a were upregulated in the EVs of HF patients following acute MI and were closely associated with left ventricular diastolic size, a critical indicator of cardiac remodeling in HF. These microRNAs, which are responsive to the tumor suppressor p53, may serve as predictive markers for future ischemic HF and cardiac remodeling. The study further revealed that p53 activation directly upregulates these microRNAs by binding to their promoter regions. In cultured myoblasts, p53 activation triggered the release of miRNA-containing EVs, which in turn enhanced cell death, highlighting their potential role in post-MI pathology [61].

In addition to their involvement in oxidative stress, plasma-derived EVs carrying mitochondrial DNA (mtDNA) have been implicated in triggering inflammatory responses in chronic HF. These mtDNA-enriched EVs activate the TLR9-NF-κB pathway, leading to increased secretion of pro-inflammatory cytokines such as IL-1β and IL-8, thereby contributing to the persistent inflammation associated with HF progression [85]. This inflammatory cascade exacerbates cardiac dysfunction and underscores the role of EVs as key mediators in HF pathology [85].

In acute decompensated HF, there is a marked increase in clot-promoting endothelial cell-derived EVs, which increase thrombin production in plasma, leading to a hypercoagulable state [86]. Indeed, a study conducted by Kou et al. [86] shows that HF patients had increased levels of PS-positive EVs and blood cells compared to healthy individuals. These EVs interact with PS-positive blood cells, accelerating coagulation and increasing FXa/thrombin synthesis, ultimately leading to heightened fibrin production and a pro-thrombotic environment [86].

### 4.4. EVs and Hypertension

Hypertension is a major independent risk factor for heart, brain, and kidney diseases, and managing resistant cases remains difficult despite the existence of multiple drugs for treatment [87]. Blood pressure regulation depends on the balance between cardiac output and vascular resistance. The renin–angiotensin–aldosterone system (RAAS), sympathetic nervous system (SNS) overactivity, and endothelial dysfunction all contribute to elevated blood pressure by promoting vasoconstriction, fluid retention, and reduced vasodilation [88]. Chronic hypertension also leads to vascular remodeling, further sustaining high blood pressure through these interconnected mechanisms [89].

EVs contribute to vascular dysfunction, alter salt and water transport in the kidney, and affect RAS. Different EV subpopulations, including platelet-derived EVs, leukocyte-derived EVs, and endothelial-derived EVs, significantly contribute to endothelial dysfunction and vascular remodeling. Platelet- and leukocyte-derived EVs impair NO bioavailability, trigger inflammatory responses in endothelial cells, and disrupt cell survival and angiogenesis [90]. Indeed, research has underlined that EVs contribute to endothelial dysfunction by primarily decreasing NO production and enhancing oxidative stress. Moreover, endothelial-derived EVs have been shown to enhance oxidative stress and promote vascular inflammation, further exacerbating endothelial dysfunction. Notably, the pathological effects of EVs are intricately mediated by the activation of signaling pathways such as PI3K/Akt and MAPK, altered expression of caveolin-1, and an increase in ROS production, which collectively contribute to vascular damage and sustained hypertension [91].

Furthermore, in patients with primary aldosteronism—the most prevalent form of secondary hypertension—elevated levels of circulating EVs have been detected. These EVs overexpress transcripts for proteins linked to vascular injury, apoptosis, and inflammation, including endothelin-1 (EDN1) and caspase-1 (CASP1), both of which contribute to endothelial dysfunction and heightened cardiovascular risk [92].

Additionally, distinct EV subtypes act as potent mediators of inflammation, as demonstrated by Nomura et al. [93], who found that EVs from hypertensive patients stimulate the expression of key inflammatory genes and cytokines such as IL-1β, IL-6, IL-8, and TNF-α in both monocytes and endothelial cells. These findings suggest that EV-induced microparticle activation plays a significant role in the vascular damage associated with inflammatory disorders [93].

Recent studies suggest that EVs may also contribute to the dysregulation of key molecular pathways involved in hypertension, such as the miRNA-mediated modulation of endothelial and vascular smooth muscle cell function. Notably, miRNAs such as miR-155 and miR-92a, enriched in EVs, have been implicated in endothelial dysfunction, oxidative stress, and inflammation [94]. These EV-associated miRNAs can suppress endothelial nitric oxide synthase (eNOS) expression, thereby reducing NO production and exacerbating vasoconstriction. Additionally, EVs derived from hypertensive patients have been shown to carry pro-inflammatory and oxidative stress-inducing molecules that contribute to vascular remodeling and fibrosis, key processes in sustained hypertension. Targeting EV-associated miRNAs through inhibitors or miRNA-mimic therapies presents a novel strategy for mitigating hypertension-related endothelial dysfunction and vascular damage [94].

## 5. Extracellular Vesicles in CVD Therapy

Over recent decades, synthetic nanoparticle delivery systems have been developed to enhance the pharmacokinetic and pharmacodynamic properties of therapeutics, reduce drug toxicity, and minimize off-target effects. Among these, liposome-based nanoparticles are notable, but their clinical application faces challenges such as rapid clearance, off-target accumulation, and immune responses [95]. EVs are being explored as vehicles for targeted drug delivery, leveraging their natural homing capabilities to deliver therapeutic agents directly to damaged cardiac tissues, thereby minimizing off-target effects [95].

EVs have emerged as promising carriers for nucleic acid therapeutics, including siRNA, plasmid DNA, miRNA mimics and inhibitors, and mRNA [96]. These therapeutic agents can be loaded into EVs through endogenous or exogenous approaches. Endogenous engineering, sometimes called passive preloading, involves manipulating the parent cells that produce EVs either genetically or pharmacologically. This can be carried out by introducing synthetic molecules, miRNA-expressing plasmids, or viral vectors into EV-producing cells, which raises the intracellular concentration of desired targets that are then naturally incorporated into EVs during their formation. This method is advantageous because it maintains the structural integrity of the vesicles and avoids directly disturbing their membranes [96]. On the other hand, exogenous engineering refers to modifications made to EVs after they have been isolated. Techniques such as electroporation, sonication, freeze–thaw cycles, or chemical transfection are used to load EVs with therapeutic substances like anti-inflammatory microRNAs, siRNAs, or drugs. Furthermore, surface modification methods, including conjugating targeting ligands or fusing EV membranes with specific peptides, can be applied to guide EVs precisely to damaged heart tissue or inflamed blood vessels. This targeted delivery enhances treatment effectiveness while reducing unintended effects elsewhere [97].

For example, in the study conducted by Zhu et al. [98], EVs derived from MSCs loaded with miR-214 demonstrated significant improvements in therapeutic effects for MI by promoting cardiomyocyte survival and enhancing EC function [98]. Additionally, MSC-derived EVs modified with CD47, a membrane surface modification that reduces immune clearance, were loaded with miR-21, which is shown to alleviate cardiac inflammation and improve myocardial ischemia/reperfusion injury outcomes. The CD47 modification prevented the EVs from being taken up by macrophages, enhancing their retention in the target tissue [99].

Moreover, EVs from adipose-derived stem cell loaded with miR-93-5p have been demonstrated to mitigate myocardial damage in MI by targeting autophagy-related proteins and inflammatory pathways, thus reducing inflammation and inhibiting autophagy in heart tissue. This dual-action approach addresses both autophagy and inflammation to enhance myocardial repair [100].

Recent studies highlight the therapeutic potential of exosomes derived from cardiac progenitor cells (CPCs) [101]. CPC-derived EVs have demonstrated the ability to enhance endothelial cell migration and protect against ischemia/reperfusion injury. Notably, EVs engineered from THP-1 monocytes expressing miR-150 have been shown to reduce expression of the miR-150 target gene c-Myb, thereby promoting endothelial migration. Further optimization using chemically stabilized miR-143 has improved EV stability and function [102].

Collectively, stem cell-derived EVs act as potent mediators of intercellular communication and hold significant promises as cell-free therapeutics for cardiovascular diseases, paralleling the regenerative capabilities of their parent cells [102].

However, one of the challenges in using EVs as drug delivery vehicles is their low loading efficiency for large therapeutics, such as RNA [103]. To address this issue, Sutaria et al. [104] developed a modified precursor miR-199a sequence to enhance RNA loading efficiency. Despite these enhancements, the study faced significant challenges, including low miRNA copies per EV, limited uptake by cells, and ineffective target gene modulation. Additionally, once internalized by recipient cells, EVs were rapidly degraded or trapped in the endosomal pathway, limiting the cytoplasmic release of their cargo [104]. This degradation, likely due to the trafficking of EVs into lysosomal pathways mediated by surface membrane proteins, significantly hampered their therapeutic efficacy. Even when RNA was successfully delivered, the amount of mature miRNA was insufficient to modulate target gene expression effectively [104]. Strategies such as engineering EV surface proteins to promote endosomal escape and modifying RNA cargo structures to enhance stability and activity could improve therapeutic outcomes [105]. Additionally, optimizing EV production and isolation methods may increase RNA yield and stability. These efforts will be crucial in unlocking the full potential of EVs as therapeutics [105].

## 6. Conclusions

This review highlights the critical role of EVs in CVD and their potential as novel biomarkers and therapeutic agents. Given the increasing global burden of CVD and the limitations of current diagnostic tools, EVs offer a promising alternative due to their ability to carry bioactive molecules that reflect disease progression. Their presence in various body fluids makes them accessible for non-invasive diagnostics, while their molecular cargo—including proteins, lipids, and miRNAs—provides valuable insights into disease mechanisms.

Despite their potential, several challenges must be addressed before EVs can be effectively implemented in clinical practice. The lack of standardized methods for EV isolation, characterization, and quantification remains a major obstacle affecting reproducibility and reliability across studies. Additionally, more extensive human studies are needed to validate the clinical utility of EVs in different stages of CVD and to establish their role in disease prediction and monitoring.

Overcoming these obstacles could pave the way for EVs to revolutionize cardiovascular medicine by enabling early detection, personalized treatment strategies, and continuous disease monitoring. Future research should focus on refining EV-based technologies, improving standardization protocols, and expanding clinical trials to fully harness their diagnostic and therapeutic potential in CVD management.

## Figures and Tables

**Table 1:** Circulating biomarkers that are currently employed in the clinical diagnosis of CVDs.

Circulating Biomarkers	Release Time	Disease	Half Time	Normal Range	Refs.
cTn-I and cTn-T ^1^	3–4 h	Endocarditis, myocardial infarction, myocarditis, pericarditis	7–10 days	≤0.04 ng/mL	[7]
BNP ^2^ and NT-proBNP ^3^	1.5–2 h	Atrial fibrillation, heart failure, stroke, transient ischemic attack	5–7 days	≤125 pg/mL	[8]
CK-MB ^4^	3–4 h	Cardiac trauma, heart transplantation, myocardial infarction, myocarditis, pulmonary embolism	24–72 h	10 to 25 IU/L	[8]
CRP ^5^	12 h	Coronary heart disease, ischemic stroke	24 h	≤0.3 mg/dL	[9,10]
Myoglobin	3 h	Acute myocardial infarction, acute myocarditis	5 min	50–85 Ug/L	[8]
D-dimer	2 h	Deep venous thrombosis, pulmonary embolism	8 h	≤0.50 mg/L	[11,12]
Homocysteine	6–8 h	Thrombosis	3–4 h	≤10 µmol/L	[13]

^1^ Cardiac troponin I and T; ^2^ brain natriuretic peptide; ^3^ N-terminal pro-B-type natriuretic peptides; ^4^ creatine kinase-MB; ^5^ C-reactive protein.

**Table 2:** Principal markers to distinguish the origin of bloodstream EVs.

EV Origin	Marker	Location	Refs.
Cardiomyocyte	Caveolin-3, flotillin-1	Membrane-embedded	[34]
Endothelial cell	CD31 (PECAM-1), CD144 (VE-cadherin), CD146 (MCAM), CD62E (E-selectin),	Surface	[35]
	von Willebrand Factor (vWF)	Intravesicular	
Erythrocyte	CD55, CD59, CD235a (Glycophorin A)	Surface	[36]
	Hemoglobin (α and β chains),	Intravesicular	
	Band 3 (Anion Exchanger 1)	Membrane-embedded	
T helper cells	CD4, CD45, CD28, CD16	Surface	[37]
T cytotoxic cells	CD8, CD45, CD28, CD16	Surface	[37]
B cells and Natural killer cells	CD49d	Surface	[37]
Monocyte	CD11b, CD14, CD64, CD142, HLA-DR	Surface	[37,38]
Neutrophil	CD35, CD66b	Surface	[39]
Platelet	CD41a (Integrin αIIb), CD42b (Glycoprotein Ib; GPIb), CD61 (Integrin β3), CD62P (P-selectin)	Surface	[40,41]

**Table 3:** Key cargo carried by EVs implicated in CVDs.

Disease	Cell Origin	EV Cargo	Clinical Outcomes	Species	Ref.
AS ^1^	Macrophages	miR-4532	Promote endothelial cell dysfunction	Human	[56]
AS	Macrophages	TNF-α	Propagation of inflammatory signals	Human	[43]
AS	Endothelial cells	CXCL-10	Promote endothelial cell dysfunction	Human	[57]
AS	Endothelial cells	ICAM-1, VCAM	Recruitment of leukocytes	Human	[58]
AS, MI ^2^	Macrophages	miR-155	Suppression fibroblast proliferation and promotion of inflammation	Human	[59]
Coronary AS	N/A	miR-30, miR-92a	Regulation of cellular cholesterol and phospholipid homeostasis	Human	[60]
HF ^3^	Myoblasts	miR 192, miR 194, and miR-34a	Induction of ventricular remodeling	Human	[61]
HT ^4^, AS	Leukocytes and platelets	IL-1, IL-8	Promotion of inflammatory responses in endothelial cells	Human	[57]
MI	N/A	miR-30a	Regulate autophagy	Human	[62]
MI	N/A	Ceramides	Induction of cardiomyocyte apoptosis and inflammation	Human	[63]
MI	N/A	Sphingolipids	Modulation of endothelial function and inflammation	Human	[63]
MI	N/A	PIgR ^5^, Cystatin C, and Complement factor C5a	Promote inflammatory responses	Human	[64]
MI, AS	Cardiomyocytes	CCL2, CCL7	Promotion of inflammatory responses	Human	[57]
MI, HT	Leukocytes, platelet, and cardiomyocytes	IL-6	Promotion of inflammatory responses	Human	[57]

^1^ Atherosclerosis; ^2^ myocardial infarction; ^3^ heart failure; ^4^ hypertension; ^5^ polymeric immunoglobulin receptor.

## Data Availability

Data sharing is not applicable.
